# Closed polymer containers based on phenylboronic esters of resorcinarenes

**DOI:** 10.3762/bjnano.9.151

**Published:** 2018-05-29

**Authors:** Tatiana Yu Sergeeva, Rezeda K Mukhitova, Irek R Nizameev, Marsil K Kadirov, Polina D Klypina, Albina Y Ziganshina, Alexander I Konovalov

**Affiliations:** 1 A. E. Arbuzov Institute of Organic and Physical Chemistry, Kazan Scientific Center, Russian Academy of Sciences, Arbuzov str. 8, Kazan 420088, Russia; 2 Kazan National Research Technical University, K. Marx str. 10, Kazan 420111, Russia; 3 A. M. Butlerov Institute of Chemistry, Kazan Federal University, Kremlevskaya str. 18, Kazan 420008, Russia

**Keywords:** boronic acid, polymer nanocontainer, resorcinarene, responsive release

## Abstract

Novel polymer nanospheres (p(SRA-B)) were prepared by cross-linking a sulfonated resorcinarene (SRA) with phenylboronic acid. p(SRA-B) shows good stability in water and can be used as a nanocontainer for the pH- and glucose-controlled substrate release. Fluorescent dyes (fluorescein, pyrene and 1,3,6,8-pyrenetetrasulfonic acid tetrasodium salt) were successfully loaded into p(SRA-B). The release of dye is achieved by lowering the pH value to 3 or by adding glucose.

## Introduction

Boronic acids effectively interact with diols [1–4] that have found application in various fields: in the recognition and sensing of carbohydrates and glycoproteins [5–11], in separation techniques [12] and in labeling and manipulation of proteins [13–15]. In recent years, boronic acid derivatives were applied in the construction of functional materials, i.e., stimuli-responsive devices and carriers for drug delivery [16–22]. In general, the boronate functional systems have a complex structure and consist of polymeric materials connected with reversible boronic ester bonds [23–27]. The external stimuli influence the formation and dissociation of the boronic bond, which leads to the reconfiguration of the systems [28–31]. The synthesis of such systems is a multi-stage process that requires high amounts of reagents and time, which inhibits significantly a practical application.

We propose a simple and easy method for the synthesis of stimuli-responsive materials. The method is based on the self-assembly of resorcinarenes, macrocyclic compounds obtained by the condensation of resorcinol with aldehydes [32]. Resorcinarenes offer the advantages of low toxicity and simple synthesis with the use of available reagents. One of their important features is the ability to self-assemble. Due to the relatively flexible structure and preorganized functional groups, resorcinarenes form a variety of three-dimensional ensembles, both in solution and in the solid state [33–35]. For the synthesis of the responsive systems, the resorcinarenes ensembles are first constructed and then their tails connected by stimuli-responsive linkers to form a closed polymeric structure. The external stimulus acts on the linker, changing the structure and functionality of the system. This approach is quite simple and opens great opportunities. Employing resorcinarenes with different functional groups and various stimulus-responsive fragments allows one to create diverse responsive systems. Earlier we constructed and reported thermo- and redox-responsive nanocontainers based on the resorcinarene cavitand [36–37]. Continuing these works we designed a new nanocontainer using resorcinarene. The container (p(SRA-B)) consists of sulfonated resorcinarenes (SRA) interconnected by phenylboronic ester bonds. The decrease of the pH value or the addition of glucose affects the boronate bond causing its dissociation and disintegration of the container. Herein, we report the synthesis of p(SRA-B) and its application in the pH- and glucose-controlled substrate release.

## Results and Discussion

**Scheme 1:**
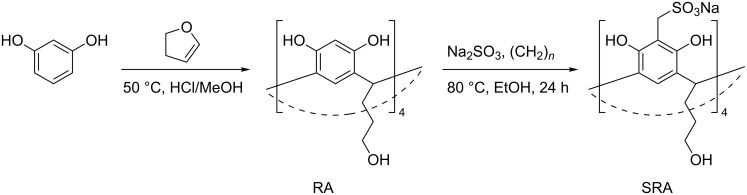
Synthesis of SRA.

Resorcinarene SRA (Scheme 1) has been synthesized in two stages. The first stage was a condensation of resorcinol with 2,3-dihydrofuran to form a macrocycle [38]. In the second stage, methylsulfonate groups were introduced at the upper rim of the macrocycle using the procedure described previously [39].

**Scheme 2:**
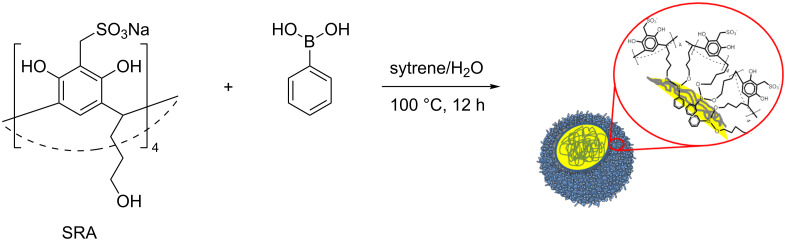
Synthesis of p(SRA-B).

**Figure 1:**
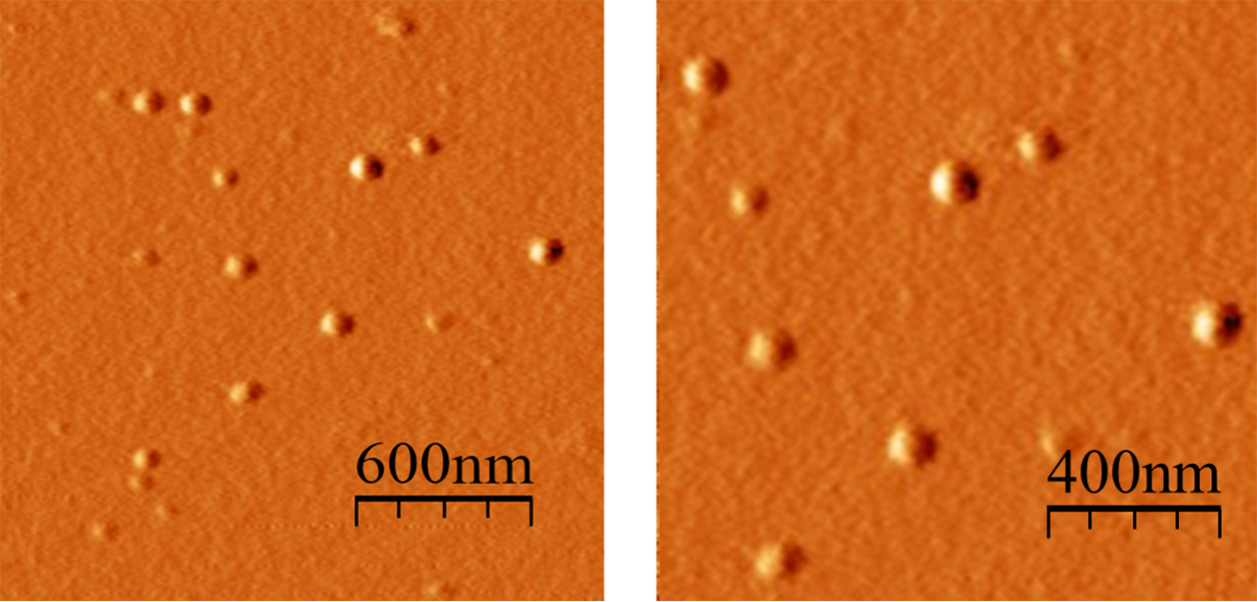
AFM images of p(SRA-B).

The microemulsion method was applied to obtain p(SRA-B). Mixing an aqueous solution of SRA (2.5 mM), phenylboronic acid (BA, 5 mM) with styrene (0.5–3 vol %) and potassium carbonate (pH 10.2) results in the formation of a microemulsion in which SRA and BA are located at the oil–water interface (Scheme 2). The charged sulfonate groups of SRA are directed into the water phase while the tails in the low rim are pointed to BA in the oil phase. The microemulsion was held for 12 h at 100 °C in order to bond SRA with BA and to form closed polymer particles. The size and dispersity of the particles depend on the volume of styrene used. Styrene and water in the ratio 1:40 (2.5 vol %) yields to the most monodisperse and uniform particles with a hydrodynamic diameter of about 200 nm and a polydispersity index (PDI) of 0.23 according to dynamic light scattering (DLS) data (see Supporting Information File 1, Figure S1, Table S1). Hereafter, we studied only the particles formed using this ratio. The nanoparticles were purified by dialysis, washed with acetone and dried to give p(SRA-B) with 66% yield. The diameter of p(SRA-B) is about 130 nm as evident from atomic force microscopy (AFM) images (Figure 1). The average molecular weight determined by static light scattering (SLS) measurements, is about 1600 ± 90 kDa (see Supporting Information File 1, Figure S2).

In the IR spectrum of p(SRA-B), the band attributed to the vibration of B–O ester bond at 1365 cm^−1^ confirms the boronate bonding between BA and SRA (see Supporting Information File 1, Figure S3). Besides, the stretching vibrations of C–H bonds at 3000–2800 cm^−1^ and the C–C aromatic bonds at 1525–1475 cm^−1^ as well as the deformational vibrations of C–C and C–H bonds at 1191, 1048 and 533 cm^−1^ are present in the IR spectrum of p(SRA-B) (see Supporting Information File 1, Figure S3). In the ^13^C and ^1^H NMR spectra of p(SRA-B), the signals of SRA, BA ester and polystyrene are present (see Supporting Information File 1, Figure S4 and Figure S5). The integrals of proton signals in the ^1^H NMR spectrum at 1.0–4.5 ppm indicate that p(SRA-B) contains one polystyrene unit per one SRA fragment, i.e., the polystyrene fraction is about 11 wt % of p(SRA-B).

According to differential scanning calorimetry (DSC) data, p(SRA-B) is thermally more stable than SRA. The DSC curve of SRA exhibits two differential peaks at 100 and 310 °C. The first peak corresponds to water loss. The second one is the thermal decomposition of SRA. In contrast to SRA, the second small peak appears only at 400 °C in the DSC profile of p(SRA-B) (see Supporting Information File 1, Figure S6). The weight of p(SRA-B) slowly decreases by 4% with the increase of temperature in the range of 250–375 °C.

The p(SRA-B) dispersion is steady in water because of the negatively charged surface formed by sulfonated groups. The ζ-potential of p(SRA-B) is −50 mV.

UV–vis spectroscopy was applied to investigate the stability and acid–base properties of SRA and p(SRA-B). For resorcinarene SRA, the UV spectrum does not exhibit significant changes when the pH value is increased from pH 2 to pH 6 (see Supporting Information File 1, Figure S7). Important changes were observed when the pH value was increased from 6.5 to 12, at which the dissociation of O–H protons occurs. SRA shows an absorbance band at 286 nm, which decreases with increasing pH value of the medium. The deprotonated forms of SRA exhibit new bands of phenolate anions at 305 and 352 nm [40]. The deprotonation constants p*K*_a_, calculated using UV–vis titration data [41], are shown in Table 1. The first and second constants of SRA are much smaller than those of resorcinol [40] because of the intramolecular hydrogen bond between hydroxy groups [42–43]. In contrast to SRA, the changes in the UV–vis spectrum of p(SRA-B) start at pH 3 and the first inflection point of the titration curve appears at pH 4.9. This curvature change is absent in the curve of SRA and refers to the dissociation of the boronic ester bond (p*K*_diss_). The p*K*_a_ values of the deprotonation of the hydroxy groups are greater for p(SRA-B), because the high density of negative charge formed by the sulfonated groups hinders the dissociation of the hydroxy groups of p(SRA-B). The titration data show that the p(SRA-B) is stable in the pH value range of 6–12, at which only the deprotonation of the upper rim occurs. When the pH is lowered from 6 to 3, the boronate bonds dissociate and p(SRA-B) breaks down.

**Table 1:** The determined p*K* values of the stepwise deprotonation of SRA (0.1 mM) and p(SRA-B) (0.14 mg/mL, which corresponds to 0.1 mM of SRA) and the dissociation of p(SRA-B) (universal buffer, 25 °C).

	p*K*_diss_	p*K*_a1_	p*K*_a2_	p*K*_a3_	p*K*_a4_

resorcinol^a^	—	9.2	10.9	—	—
SRA	—	7.2	8.7	11	11.4
p(SRA-B)	4.9	8.2	10.0	11.1	12.2

^a^Data are taken from [40].

The boronic ester bonds are dynamic and reversible [44]. They undergo transesterification with different alcohols but preferably interact with 1,2-*cis*- and 1,3-diols to produce stable five- and six-membered rings. Such diols include glucose, which displaces alcohol moieties to form the glucose boronic ester [45]. DLS data show that indeed glucose destroys p(SRA-B). When glucose is added to the p(SRA-B) solution, the PDI increases from 0.23 to 0.47, and multiple peaks appear in the range of 40–1000 nm (see Supporting Information File 1, Figure S8).

p(SRA-B) was examined as a container for pH- and glucose-controlled storage and as a source of substrates. Three dyes were chosen: fluorescein (Fl), pyrene (Py) and 1,3,6,8-pyrenetetrasulfonic acid tetrasodium salt (PTS). These dyes differ in polarity but all of them are used as fluorescent probes to determine the environments solvent [46]. It is possible to control the location of the dyes in the container as well as their release when the container is destroyed, using fluorescence spectroscopy.

**Figure 2:**
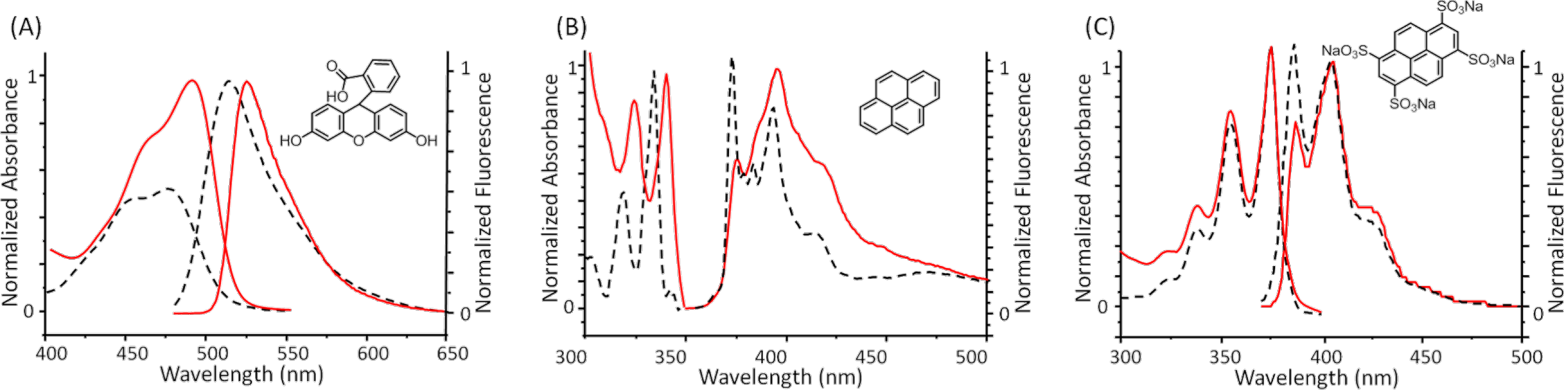
UV–vis and fluorescence spectra of (A) Fl, (B) Py and (C) PTS in aqueous media (black dashed line) and encapsulated in p(SRA-B) (red solid line) (0.27 mg/mL, which corresponds to 0.2 mM of SRA, 25 °C).

The synthesis of the nanoparticles with the dyes (D@p(SRA-B), where D = Fl, Py and PTS) was carried out similar as p(SRA-B) but using aqueous solutions of the dyes (5 mM) instead of water. After the synthesis, D@p(SRA-B) were dialyzed for three days. The encapsulation efficiency (*EE*) of p(SRA-B) toward PTS and Fl is 9.3 and 9.6%, respectively (Table 2). Due to the poor solubility of Py in water and the tendency to aggregate, precise quantification of pyrene is difficult. The analysis of the UV and fluorescent spectra of D@p(SRA-B) showed that all of the used dyes are encapsulated in the cavity of p(SRA-B) despite the difference in their polarity. For Fl@p(SRA-B), a bathochromic shift of the absorption and emission bands indicates the location of Fl in the organic phase [47–48] (Figure 2). Py@p(SRA-B) exhibits a bathochromic shift of the absorption bands relative to free Py. In the fluorescence spectrum of Py@p(SRA-B), the decrease in the intensity of the first emission band in comparison with the second and the third bands reveals the hydrophobic nature [49–50] of the p(SRA-B) core. A similar picture is observed for PTS@p(SRA-B). The ratio of the first and the third emission band II/IIII is much lower for PTS@p(SRA-B) than for free PTS [51] (Figure 2). SRA itself hardly interacts with the used dyes and does not significantly affect their optical characteristics. As it is evident from Figure S9 (Supporting Information File 1), the dye spectra practically do not change in the presence of 10-fold excess of SRA.

**Table 2:** Encapsulation efficiency of p(SRA-B) toward D (D = Fl and PTS) and release of D from D@p(SRA-B) (0.27 mg/mL) at pH 3 (universal buffer), and after addition of glucose (0.4 mM), 25 °C.

D	*EE*, %	release, %	

		pH 3	glucose

Fl	9.6	44	90
PTS	9.3	47	99

**Scheme 3:**
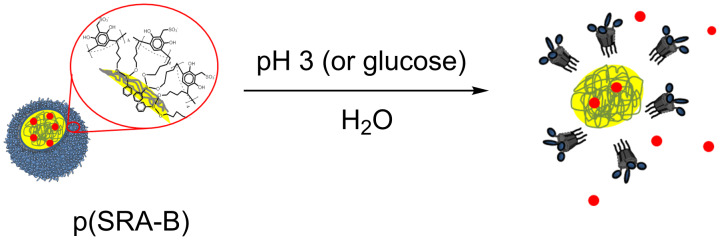
Dye release from p(SRA-B) under the action of pH value or glucose.

**Figure 3:**
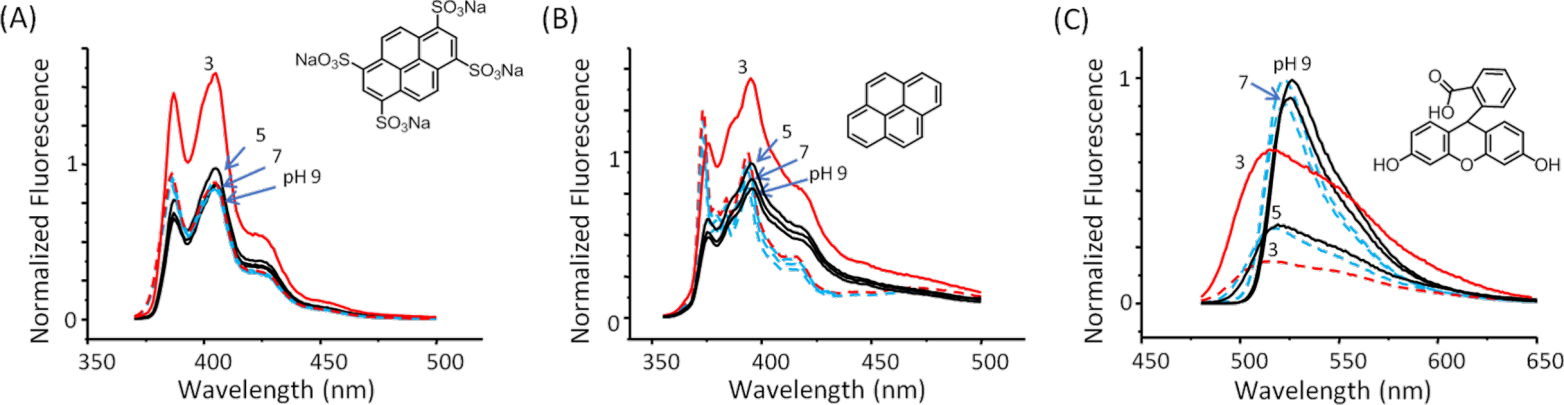
Fluorescence spectra of free D (dashed lines) and D@p(SRA-B) (0.27 mg/mL) (solid lines) at pH 3–9 where D is (A) PTS, (B) Py and (C) Fl (universal buffer, 25 °C).

D@p(SRA-B) are stable in water and no D was released over a time of more than three weeks as evident from fluorescence spectroscopy data. However, when the solution is acidified, p(SRA-B) dissociates and the dyes are released into the water (Scheme 3). The fluorescence spectra of PTS@p(SRA-B) do not change when the pH value is decreased from 9 to 5, but the emission increases at pH 3 (Figure 3). Moreover, the intensity of the first peak becomes significantly higher, which indicates the release of PTS from the organic environment into the water. For Py@p(SRA-B), an increase in the emission intensity is also observed, confirming the destruction of the container. However, the shape of the fluorescence spectrum remains practically unchanged. Apparently, Py is not released into the water, it remains in the aggregated state in supramolecular associates formed after the dissociation of p(SRA-B). In the case of Fl@p(SRA-B), both shape and intensity of the fluorescence spectrum are changed upon acidification. As it is known, Fl is a pH-sensitive fluorescent dye. Its fluorescence decreases during the switch from alkaline to acidic media. The same happens for Fl@p(SRA-B). A decrease of the pH value to 5 results in a decrease in emission intensity of Fl@p(SRA-B) (Figure 3). However, at pH 3 the fluorescence peak increases and there is a hypsochromic shift due to the release of Fl.

To determine the release of D (D = Fl, PTS) after acidification, dialysis was carried out for three hours and the concentration of D in the dialysate was monitored using UV spectroscopy (Table 2). Indeed, p(SRA-B) is decomposed at pH 3 and 44–47% of the dye molecules are released from the cavity into the solution.

**Figure 4:**
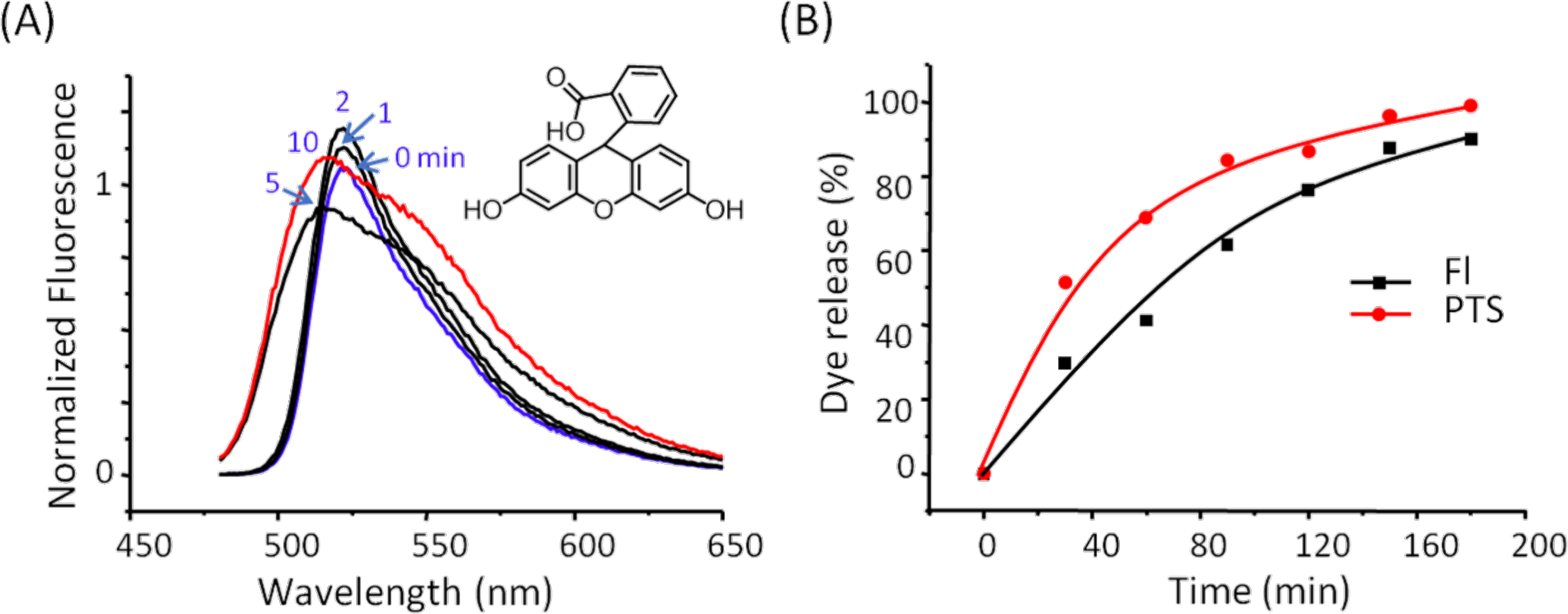
(A) Fl@p(SRA-B) spectra after addition of glucose (0.4 mM); D release from D@p(SRA-B), where D = Fl, PTS, after addition of glucose (0.4 mM) and dialysis (H_2_O, 25 °C).

The dissociation of the capsule can also occur after the addition influence of glucose. When glucose is added to the aqueous solution of Fl@p(SRA-B), a slight increase in the fluorescence emission intensity is observed in the first two minutes (Figure 4). Then a hypsochromic shift of the fluorescence band occurs in the next 5–10 minutes (Figure 4). To determine the quantitative yield, D@p(SRA-B) (D = Fl, PTS) were dialyzed (Table 2, Figure 4). Almost all of the dye was released into water within three hours of dialysis (Table 2, Figure 4).

## Conclusion

Closed polymer nanoparticles p(SRA-B) were successfully prepared by cross-linking sulfonated resorcinarene (SRA) with phenylboronic acid (BA). p(SRA-B) exhibits good stability over a wide range of pH (6–12). In acidic media, p(SRA-B) dissociates after breaking of the B–O bonds between SRA and BA. This feature can be used for a pH-controlled substrate release. Three dyes Fl, Py and PTS were successfully loaded into the p(SRA-B) cavity. The dyes were not released from p(SRA-B) over a period of more than three weeks in water. However, reducing the pH value down to 3 results in the dissociation of p(SRA-B) and a rapid release of the dyes. A similar behavior was observed after the addition of glucose.

## Experimental

### Characterizations

An atomic force microscope (AFM, Innova, Bruker) has been used to reveal the morphology of the nanoparticles. Cantilevers with silicone tips (250–350 kHz, tip curvature radius of 10–12 nm, Veeco) have been used in all measurements. The images were obtained with a resolution of 512 × 512 pixels. The scanning rate was 1 Hz. The antivibrational system (SG0508) has been used to eliminate external distortions. The tip-convolution effect has been minimized by processing the obtained AFM data with the use of WSxM 5.0, Zod 2.0 and MatLAB software [52–53]. The calibration has been performed by using an imaging special calibration grid (STR3-1800P, VLSI Standards Inc.) in the temperature range of 20–60 °C. A Zetasizer Nano instrument (Malvern, UK) equipped with a 4 mW He:Ne solid-state laser operating at 633 nm was used for SLS and DLS experiments, and ζ-potential measurements. Malvern dispersion technology software 5.0 was used for the analysis of particle size, ζ-potential, and molecular weight. IR spectra were recorded in the region of 4000–400 cm^−1^ by using a Bruker Vector-22 FTIR-spectrophotometer with a resolution of 4 cm^−1^. Samples for the registration of the IR spectra were prepared in KBr pellets. NMR spectroscopic experiments were carried out with an Avance 600 spectrometer (Bruker, Germany) equipped with a pulsed gradient unit capable of producing magnetic-field pulse gradients in the *z*-direction of about 56 G/cm. D_2_O was used as solvent in all experiments. Chemical shifts were reported relative to H_2_O (δ = 4.7 ppm) as an internal standard. Measurements by the DSC and TGA methods were carried out with a Netzsch STA 449C Jupiter instrument in argon in the temperature range from 30 to 200 °С. The heating rate was 10 °C·min^−1^. The samples were placed in aluminum crucibles (non-sealed for the free removal of evolved products and a decrease of the influence of an excessive pressure). UV–vis spectra were recorded with a Perkin–Elmer Lambda 25 UV–vis spectrometer. А cuvette with an optical path length of 50 mm was used in all experiments. The pH value was measured with a pH meter Orion 2STAR pH benchtop (Thermo Scientific). Fluorescence emission spectra were recorded with a Cary Eclipse fluorescence spectrophotometer. A quartz cell of 1 cm path length was used for all fluorescence measurements. Fl, Py and PTS were excited at 461, 337 and 360 nm, respectively.

### Chemicals

Resorcinol, Na_2_SO_3_, paraldehyde, St, BA, Fl, Py and PTS were purchased from Sigma-Aldrich and Acros Organics. All materials were used as received without any further purification. Resorcinarene RA was synthesized as described in [38].

### Synthesis of SRA

A mixture of Na_2_SO_3_ (3.45 g, 27.4 mmol) and paraldehyde (0.823 g, 27.4 mmol) was heated in 5 mL of water at 80 °C until complete dissolution. Then RA (2 g, 2.74 mmol) in 30 mL ethanol was added dropwise. The suspension was mixed at 80 °C for 6 h. The solid was filtered and dissolved in water. HCl was added to the solution to adjust the pH value to 5–6. The solution was dialyzed (3 × 30 min) and the solvent was removed by vacuum distillation. Yellow oil was triturated in ethanol, filtered and washed with ethanol (3.15 g, 96%). ^1^H NMR (600 MHz, D_2_O) δ 7.21 (s, 4H), 4.50 (t, 4H), 4.29 (s, 8H), 3.65 (t, 8H), 2.21 (d, 2H), 1.57 (m, 2H); ^13^C NMR (600 MHz, D_2_O) δ 152, 125, 122, 108, 62, 48, 34, 30, 29; IR (cm^−1^) 3390, 2939, 2871, 1608, 1473, 1213, 1150, 1041, 979, 900, 800, 778, 760, 664, 630, 545 497; Anal. calcd for C_44_H_52_Na_4_O_24_S_4_: C, 44.59; H, 4.42; Na, 7.76; S, 10.82; found: C, 44.26; H, 4.68; Na, 7.89; S, 10.89; MS (MALDI): calcd for C_44_H_52_O_24_S_4_^4−^: 1093; found: 1095 (C_44_H_52_O_24_S_4_^4−^ + 2H^+^), 1116 (C_44_H_52_O_24_S_4_^4−^ + Na^+^), 1139 (C_44_H_52_O_24_S_4_^4−^ + 2Na^+^).

### Synthesis of p(SRA-B)

Styrene (50 μL) was added to 2 mL of an aqueous solution containing SRA (2.5 mM), BA (5 mM) and K_2_CO_3_ (0.3 mM) with pH 10.2. The mixture was bubbled with argon for 30 min then sonicated in argon atmosphere for 30 min and finally again bubbled with argon for 30 min. The reaction mixture was heated at 100 °C for 12 h. Then mixture was dialyzed against K_2_CO_3_ (7 mM) solution for 30 min and then two times against water for 30 min. Solvent was removed under reduced pressure. The formed solid was washed with acetone and dried (4.7 mg, 66%). ^1^H NMR (600 MHz, D_2_O) δ 7.7–7.0 (br), 4.6–3.8 (br), 3.8–3.2 (br), 2.4–1,7 (br) 1.7–1.0 (br); ^13^C NMR (600 MHz, D_2_O) δ 162, 131, 127, 72, 68, 62, 61, 52, 47, 30, 29; IR (cm^−1^) 3437, 2932, 1600, 1365, 1191, 1048, 839, 757, 622, 533; Anal. calcd for C_56_H_59_B_2_Na_4_O_24_S_4_·10K_2_CO_3_: C, 28.93; H, 2.17; B, 0.79; K, 28.54; Na, 3.36; S, 4.68; found: C, 28.54; H, 2.29; B, 0.87; K, 28.44; Na, 3.58; S, 4.74. Similar reactions with different volumes of styrene (10, 20, 30, 40, 50 and 60 μL) were carried out.

### Synthesis of D@p(SRA-B) (D = Fl, Py, PTS)

SRA (5.93 mg, 5 μmol) and BA (1.22 mg, 10 μmol) were dissolved in 2 mL of an aqueous solution containing D (5 mM) and K_2_CO_3_ (0.3 mM). Styrene (50 μL) was added to the mixture. The mixture was bubbled with argon for 30 min and then sonicated in argon atmosphere for approximately 60 min. The suspension was again bubbled with argon for 30 min. The reaction mixture was heated at 100 °C for 12 h. The resulting mixture was purified from the non-encapsulated dyes by dialysis. Dialysis of 2 mL of the suspension was carried out in 50 mL of distilled water for three days using a dialysis bag with a pore size of 2000 Da. The UV spectrum of the dialysate was determined in the range of 200–600 nm.

### Determination of p*K*_diss_ and p*K*_a_ values

0.3 mL of SRA (1 mM) or p(SRA-B) (1.4 mg/mL) were added to 2.7 mL of universal buffer [54] (0.04 M) with a certain pH value (see Supporting Information File 1, Table S2) in the pH range of 1.6 to 12. After that, UV spectra were measured in the range of 250–600 nm. p*K* values were calculated from changes in the absorbance at 305 and 352 nm using CurTiPot (version 4.2.3) software [41].

### pH-controlled dye release from D@p(SRA-B)

0.3 mL of D@p(SRA-B) (2.7 mg/mL) aqueous solution was diluted with universal buffer with pH 9.00, 7.05, 5.20 or 3.28 and the fluorescent spectra were recorded using a fluorescence spectrophotometer. To study the kinetics of the pH-controlled dye release, dialysis of D@p(SRA-B) (3 mL, 0.27 mg/mL) in 50 mL of universal buffer solution (pH 3) was carried out using a dialysis bag with a pore size of 2000 Da. UV spectra of the dialysate were measured every 30 min over a time span of three hours.

### Glucose-controlled release of fluorescein from D@p(SRA-B)

0.3 mL of a glucose solution (4 mM) in water and 2.4 mL of distilled water were added to 0.3 mL of D@p(SRA-B) aqueous solution (2.7 mg/mL). The fluorescent spectra were recorded after 0, 1, 2, 5 and 10 min. To study the kinetics of the glucose-controlled dye release, dialysis of the D@p(SRA-B) solution (3 mL, 0.27 mg/mL) in 50 mL of the 0.4 mM glucose solution was carried out using a dialysis bag with a pore size of 2000 Da. UV spectra of dialysate were determined every 30 min over a time of three hours.

## Supporting Information

Supporting Information features data for SRA and p(SRA-B): size distribution diagram, DLS and SLS data, IR spectra, NMR spectra, TGA and DSC data, acid-base titration curves; UV- and fluorescence spectra of D in the presence of SRA; preparation of universal buffer.

File 1 Additional experimental data.
