# Supplementary material for: Closed polymer containers based on phenylboronic esters of resorcinarenes
Source: Beilstein J Nanotechnol. 2018 May 29;9:1594–601. doi: 10.3762/bjnano.9.151 (PMC6009270; doi:10.3762/bjnano.9.151)
Supplement: File 1 — Additional experimental data. [file Beilstein_J_Nanotechnol-09-1594-s001.pdf]

Supporting Information

for

Closed polymer containers based on phenylboronic esters of resorcinarenes

Tatiana Yu. Sergeeva[1], Rezeda K. Mukhitova[1], Irek R. Nizameev[1,2], Marsil K. Kadirov[1], Polina D. Klypina[3], Albina Y. Ziganshina*[1,3] and Alexander I. Konovalov[1,3]

Address: [1]A. E. Arbuzov Institute of Organic and Physical Chemistry, Kazan Scientific Center, Russian Academy of Sciences, Arbuzov str. 8, Kazan 420088, Russia, [2]Kazan National Research Technical University, K. Marx str. 10, Kazan 420111, Russia and [3]A. M. Butlerov Institute of Chemistry, Kazan Federal University, Kremlevskaya str. 18, Kazan 420008, Russia

Email: Albina Y. Ziganshina - az@iopc.ru

*Corresponding author

Additional experimental data

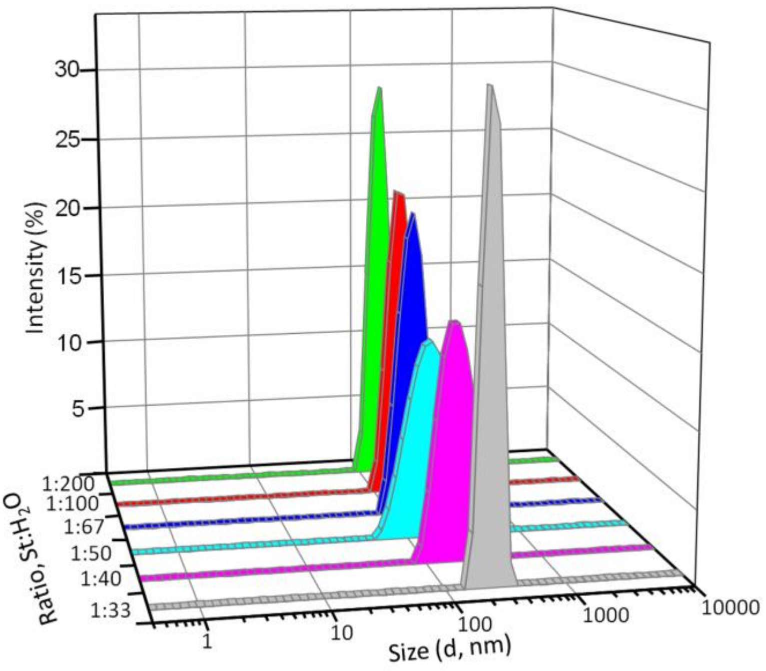

Figure S1: Size distribution diagrams of polymer nanoparticles obtained by polymerization of SRA (2.5 mM) with BA (5 mM) at a different volume ratio of styrene:H_2O.

Table S1: Dynamic light scattering data for particles formed by microemulsion polymerization of SRA (2.5 mM) with BA (5 mM) at a different ratio of styrene:H_2O; 25 °C.

Volume ratio, St:H_2O	Average hydrodynamic diameter (nm)	Polydispersity index (PDI)
1 : 200	160	0.339
1 : 100	180	0. 306
1 : 66	165	0.390
1 : 50	170	0.348
1 : 40	200	0. 262
1 : 33	235	0.392

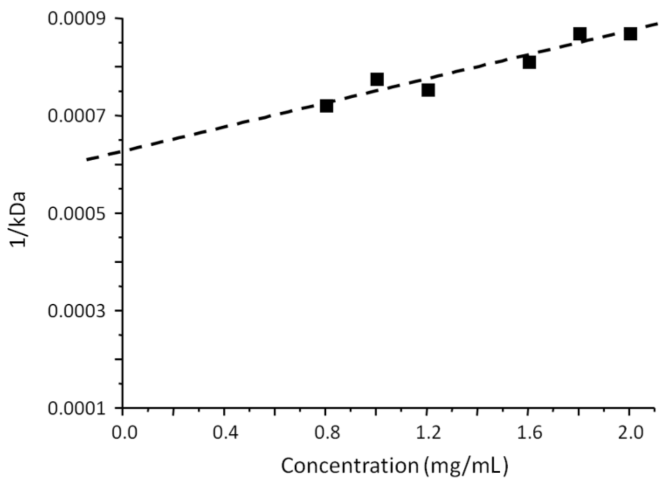

Figure S2: Debye plot for p(SRA-B), C = 0.8–2 mg/mL; H_2O.

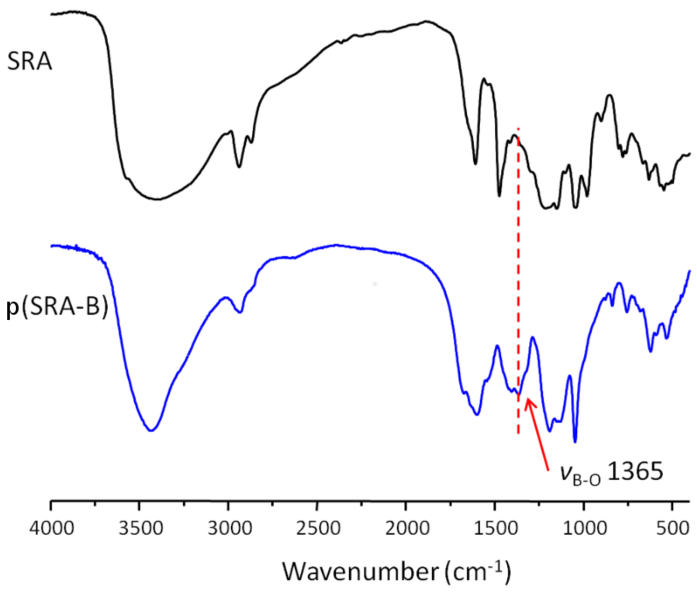

Figure S3: IR spectra of SRA and p(SRA-B).

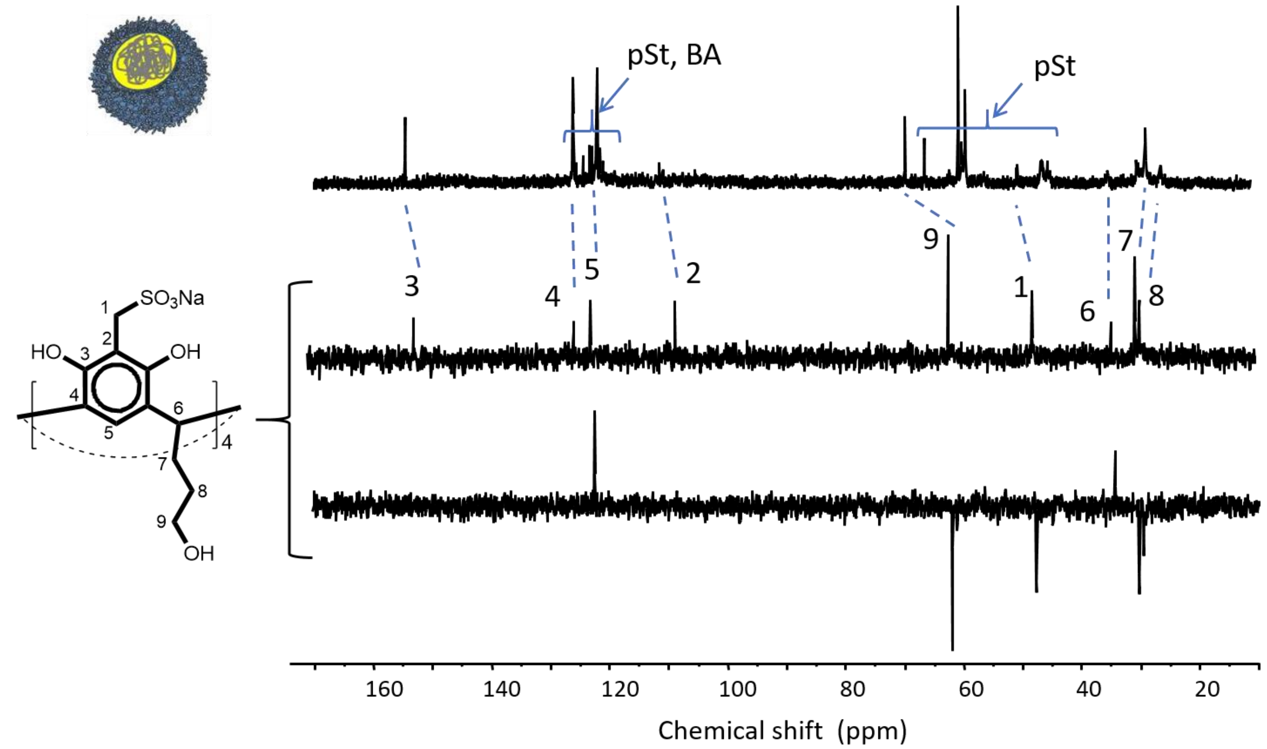

Figure S4: ^{13}C NMR spectra of SRA and p(SRA-B) in D_2O.

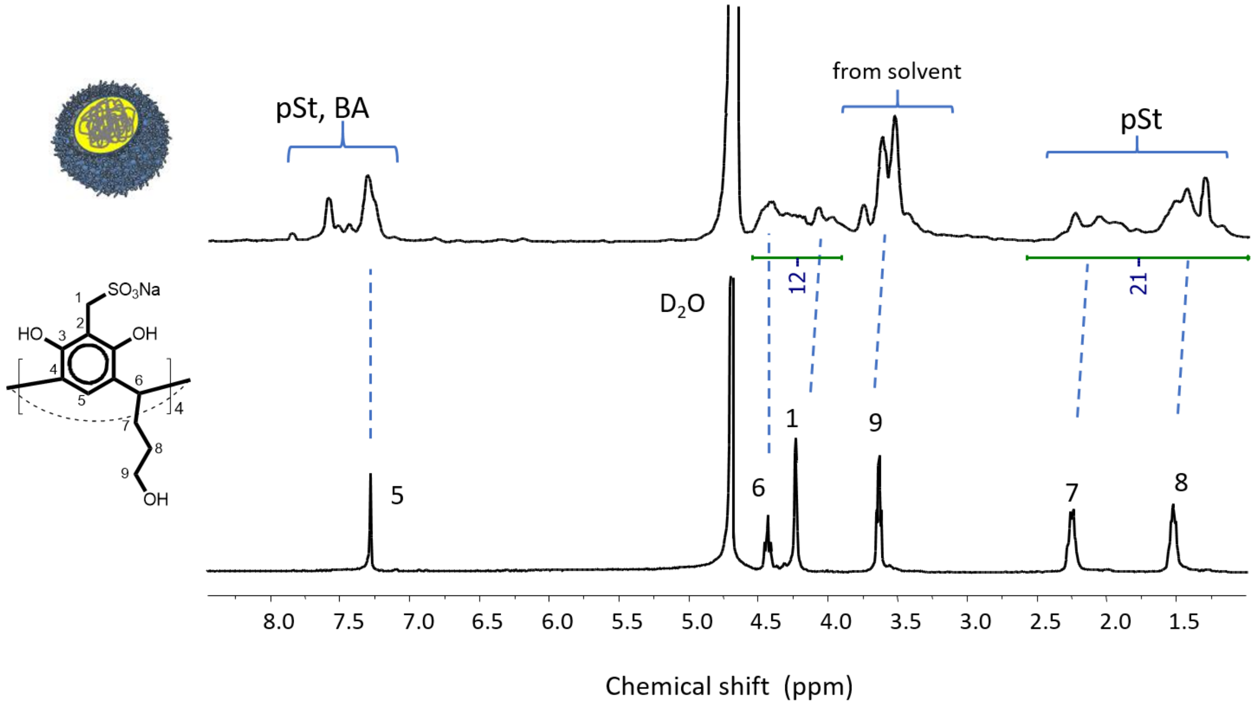

Figure S5: ^{1}H NMR spectra of SRA and p(SRA-B) in D_2O.

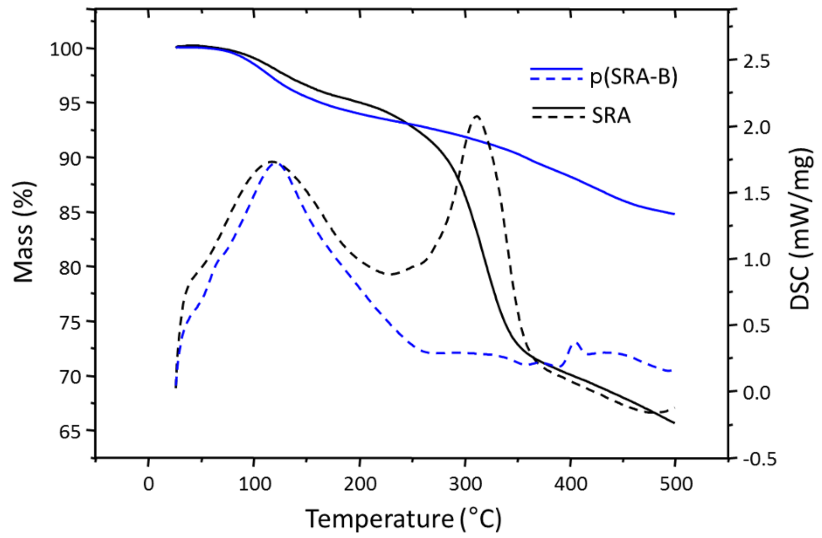

Figure S6: TGA (solid lines) and DSC (dashed lines) data for SRA and p(SRA-B).

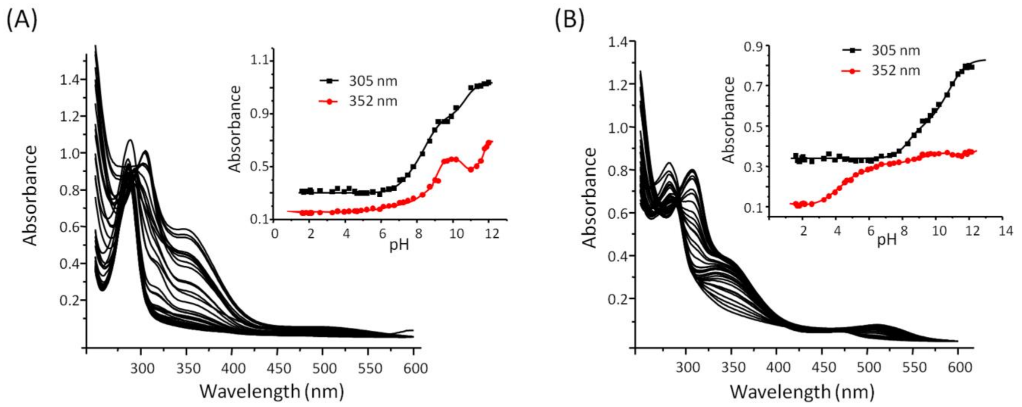

Figure S7: pH depended UV spectral change of (A) SRA (0.1 mM) and (B) p(SRA-B) (0.14 mg/mL); inside: absorbance change at 305 nm and 352 nm plotted as a function of pH (1 cm cell).

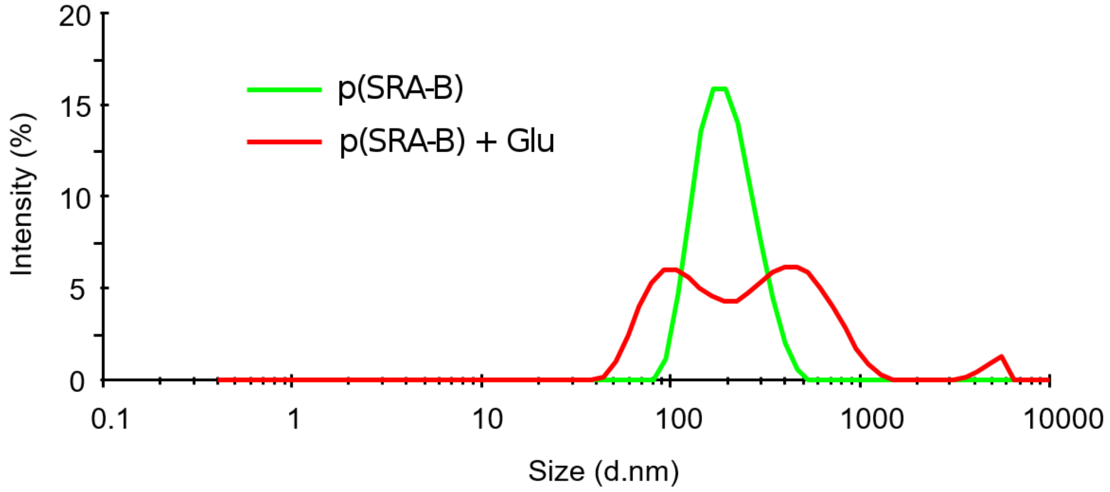

Figure S8: Size distribution diagrams of p(SRA-B) (0.27 mg/mL) before and after addition of glucose (Glu, 0.4 mM).

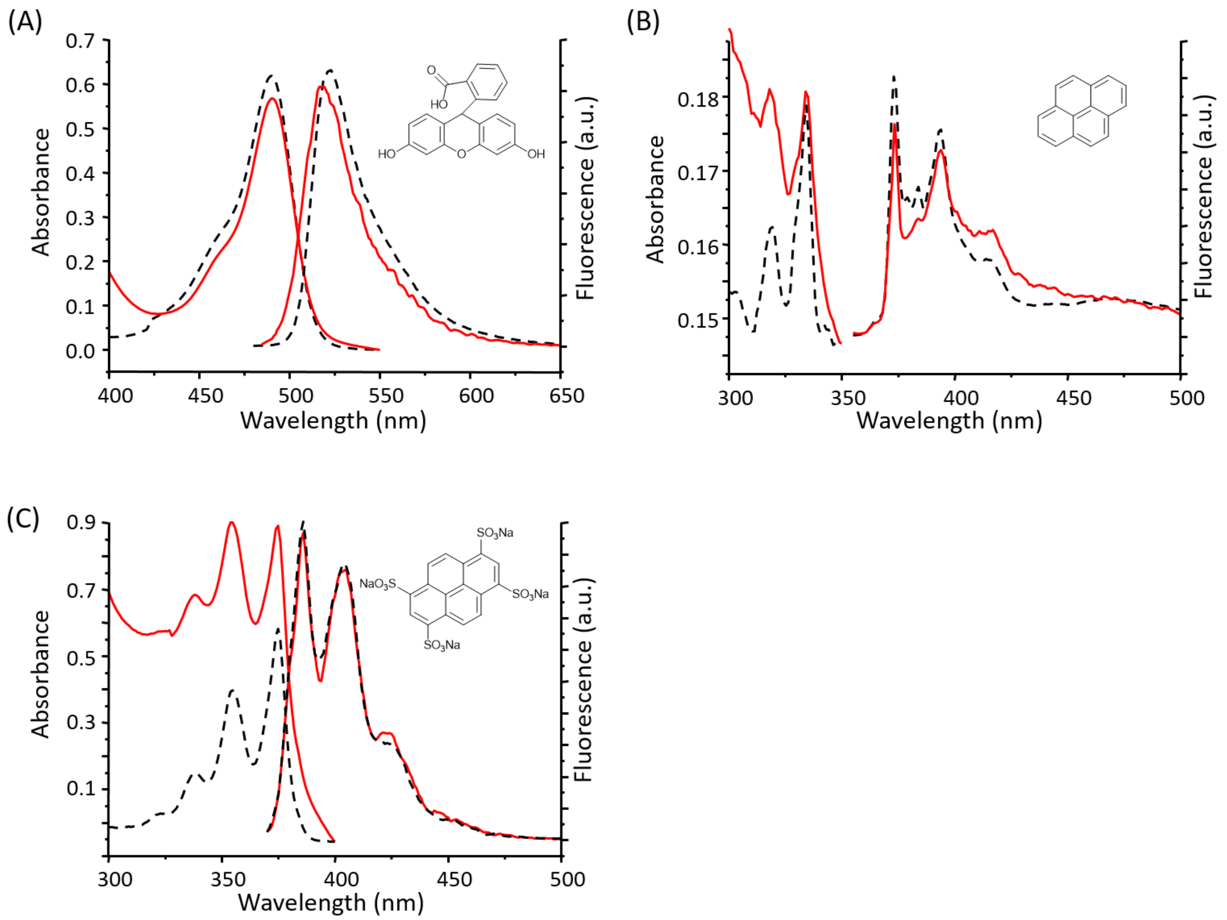

Figure S9: UV and fluorescence spectra of (A) Fl (0.01 mM), (B) Py (0.01 mM) and (C) PTS (0.01 mM) before (black dashed lines) and after (red solid lines) addition of SRA (0.1 mM), H_2O, 25 °C.

Table S2: Preparation of an universal buffer. An universal buffer was prepared as described in [1]. For this, a mixture of phosphoric (0.04 M), acetic (0.04 M) and boric (0.04 M) was prepared and then *x* mL of 0.2 M NaOH solution was added to 100 mL of the acids mixture.

NaOH, *x* mL	pH	NaOH, *x* mL	pH	NaOH, *x* mL	pH
0	1.61	37.5	5.20	72.5	9.52
2.5	1.70	40	5.54	75	9.69
5	1.82	42.5	5.93	77.5	9.92
7.5	1.96	45	6.12	80	10.19
10	2.13	47.5	6.44	82.5	10.69
12.5	2.05	50	6.73	85	11.01
15	2.60	52.5	7.05	87.5	11.34
17.5	3.28	55	7.24	90	11.59
20	3.58	57.5	7.49	92.5	11.76
22.5	3.95	60	7.80	95	11.88
25	4.22	62.5	8.32	97.5	12.00
30	4.54	65	8.71	100	12.08
32.5	4.66	67.5	9.00		
35	4.90	70	9.20		

References

1. Lurie, J. *Handbook of Analytical Chemistry;* Goskhimizdat, Moscow, Soviet Union 1962.
